# The role of uropathogenic *Escherichia coli* virulence factors in the development of urinary tract infection

**DOI:** 10.25122/jml-2024-0396

**Published:** 2025-08

**Authors:** Ketema Bizuwork Gebremedhin, Wondwossen Amogne, Haile Alemayehu, Shubhada Bopegamage, Tadesse Eguale

**Affiliations:** 1 Aklilu Lemma Institute of Pathobiology, Addis Ababa University, Addis Ababa, Ethiopia; 2 College of Health Sciences, Addis Ababa University, Addis Ababa, Ethiopia; 3 Faculty of Medicine, Slovak Medical University, Bratislava, Slovak Republic; 4 Ohio State University Global One Health, Addis Ababa, Ethiopia

**Keywords:** urinary tract infection, uropathogenic *Escherichia coli*, virulence factors, pathogenesis

## Abstract

Urinary tract infections (UTIs) are a significant global public health issue, with *Escherichia coli (E. coli)*, specifically uropathogenic *E. coli* (UPEC), being the predominant causative agent. UTIs affect millions of people annually, with a particularly high prevalence among women, children, the elderly, and individuals with compromised immunity or structural abnormalities of the urinary tract. UPEC has evolved a suite of specialized virulence factors like adhesins, flagella, capsular polysaccharides, lipopolysaccharide (LPS), outer membrane vesicles (OMVs), toxins, iron acquisition systems, autotransporters (ATs), and unique proteins such as TcpC and OmpT, that facilitate colonization, immune evasion, and disease progression. These factors enable the pathogen to cause both uncomplicated infections, such as cystitis, and more severe outcomes, including pyelonephritis and, in some cases, renal failure. The host defends against infection through mechanisms such as urine flow, urothelial shedding, cytokine release, antimicrobial peptides, and adaptive immunity. Despite advancements in medical care, the burden of UTIs remains high, underscoring the need for continued research into their pathogenesis and prevention.

## INTRODUCTION

Urinary tract infections (UTIs), defined as the presence of bacterial isolates exceeding 10^5^ colony-forming units (CFU)/mL in asymptomatic individuals and >10^2^ CFU/mL in symptomatic individuals [1], are a significant global health issue. This condition affects over 150 million people annually worldwide [2], with cases increasing from 252.25 million in 1991 to 404.61 million in 2019, a 60% rise, underscoring the growing burden of UTIs worldwide, particularly in low-income countries [3]. UTIs are the second most common infectious disease worldwide, next to upper respiratory tract infections [4], which underscores both the widespread prevalence and the significant healthcare impact of UTIs across diverse populations [4].

Urinary tract infections are clinically classified into uncomplicated and complicated cases, based on the presence of structural or neurological abnormalities in the urinary tract [1,5]. Risk factors for UTIs include being female, pregnancy, immunosuppression, genetic susceptibility, urinary obstruction, urinary retention, indwelling catheters, drainage devices, renal failure, renal transplantation, diabetes, prior UTI, vaginal infection, obesity, age, and poor hygienic practices [2]. Both Gram-negative and Gram-positive bacteria cause UTIs, as well as certain fungi [2]. The majority of urinary tract infections are caused by bacteria from the *Enterobacteriaceae* family, with uropathogenic *Escherichia coli* (UPEC) being the predominant pathogen [1,2,4]. UPEC is responsible for over 80% of uncomplicated UTIs and more than 95% of both community- and hospital-acquired infections [1,2]. Other bacterial species, such as *Klebsiella pneumoniae, Proteus mirabilis*, and *Enterobacter cloacae*, are also associated with UTIs [1,2].

## ESCHERICHIA COLI

*Escherichia coli* is a rod-shaped, Gram-negative bacillus belonging to the family Enterobacteriaceae and represents the most prevalent commensal inhabitant of the gastrointestinal tract (GIT). In its commensal form, it engages in mutually beneficial interactions with the host. However, pathogenic strains of *E. coli*, distinguished from the normal flora by their virulence factors such as exotoxins [6], are responsible for significant diseases in both humans and animals. The urinary tract is the most common site of *E. coli* infection, and more than 90% of all uncomplicated urinary tract infections are caused by *E. coli* [7,8]. *Escherichia coli* was first described by Theodor Escherich, a German pediatrician, who identified it both as a harmless intestinal inhabitant and as a pathogen capable of inducing diseases such as diarrhea, dehydration, and, in severe cases, death [9].

The bacteria can exist in either motile or non-motile states. In its motile state, it produces flagella in addition to fimbriae or pili, which are stretched outwards and play a role in attaching the cells to other cells or host tissues [10,11]. *Escherichia coli* occurs in diverse forms in nature, ranging from commensal strains to pathogenic variants infecting human/animal hosts [12]. The bacteria can survive in diverse environments, including the intestines of reptiles, birds, and mammals, water bodies, and moist soil [13]. The bacterium grows under both oxygen-rich and oxygen-limited conditions, utilizing alternative electron acceptors such as nitrate (NO_3_), nitrite (NO_2_), and fumarate under anaerobic conditions. Such versatility enables *E. coli* to thrive in anaerobic and aerobic environments [14–16].

The pathogen is opportunistic, causing disease primarily in immunocompromised hosts, and is capable of inducing severe infections such as diarrhea, dysentery, hemolytic uremic syndrome (HUS), UTIs, and septicemia. The distinction between non-pathogenic and pathogenic *Escherichia coli* was first hypothesized by Lesage in 1897, a concept later supported by Bray, who isolated enteropathogenic *E. coli* (EPEC) strains from neonates with diarrhea [15,17]. Further, Dobrindt and his colleagues compared the nonpathogenic *E. coli K-12* strain MG1655 with 26 pathogenic strains and found great heterogeneity among *E. coli* strains [18]. *E. coli K-12* was found to be missing several virulence factors as compared to its pathogenic counterparts, whereas pathogenic strains were equipped with pathogenicity islands and integrated with phage genomes [18–20]. The molecular mechanisms underlying this diversity include genomic recombination, acquisition of lysogenic phages carrying virulence genes, integration of pathogenicity islands (foreign DNA segments acquired from other bacterial species), and horizontal gene transfer via conjugation [18–20].

Phylogenetically, *E. coli* has been divided into six groups (five major), denoted as A, B1, B2, C, D, and E [12,21]. These subgroups encompass saprophytic strains (e.g., group A) and pathogenic types (in particular B2, D) and are often considered to be the result of long-term evolutionary processes [21]. Although the genetics and biochemistry of *E. coli* have been extensively studied, its behavior in natural environments, including within animal and human hosts, remains less well understood [21]. The phylogenetic groups differ in their ecological niches, life history, and biochemical characteristics [22,23]. Furthermore, groups A and B1 have comparatively smaller genome sizes when compared with the B2 and D groups. Groups A and B1 are typically commensal and persist in environmental reservoirs, whereas B2 and D strains are enriched for virulence factors (VFs) and are more frequently associated with pathogenicity [24,25].

Incontrovertibly, *E. coli* can be classified based on its virulence factors, host, and site of infection. Pathogenic *E. coli* are categorized into enteric/diarrheagenic and extra-intestinal *E. coli* [26]. Enteric pathotypes infect the intestinal mucosa, leading to dysentery, diarrhea, and related gastrointestinal disorders. In contrast, extra-intestinal pathogenic *E. coli* (ExPEC) maintain a friendly niche inside the intestine and cause infections in an extra-intestinal niche of the central nervous system (CNS), blood, respiratory tract, and urinary tract [27]. Diarrheagenic *E. coli* comprise six well-defined pathotypes: enteropathogenic *E. coli* (EPEC), enterohemorrhagic *E. coli* (EHEC), enterotoxigenic *E. coli* (ETEC), enteroaggregative *E. coli* (EAEC), enteroinvasive *E. coli* (EIEC), and diffusely adherent *E. coli* (DAEC) [11]. Extra-intestinal pathogenic *E. coli* are further classified into UPEC, meningitis-associated *E. coli* (MNEC), and avian pathogenic *E. coli* (APEC) [11].

## UROPATHOGENIC *ESCHERICHIA COLI* AND ITS VIRULENCE FACTORS

UPEC are specialized strains of *Escherichia coli* that have adapted to thrive and cause infections in the urinary tract. While *E. coli* is commonly found as part of the normal intestinal microbiota, UPEC strains are distinguished by their ability to invade and persist in the urinary tract, leading to conditions such as UTIs [28]. The bacteria are one of the major etiologic agents in causing UTIs and are responsible for 80-90% of community-acquired UTIs [2,29].

**Figure 1:**
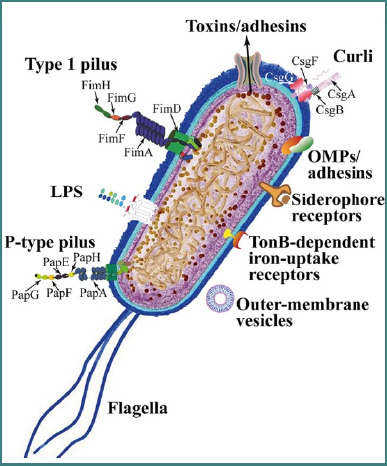
*Escherichia coli* adhesins and motile structures. Adapted from Terlizzi ME, Gribaudo G, Maffei ME. Uropathogenic *Escherichia coli* (UPEC) infections: virulence factors, bladder responses, antibiotic, and non-antibiotic antimicrobial strategies. Pathogens. 2017;6(2):E19. doi:10.3390/pathogens6020019

**Figure 2:**
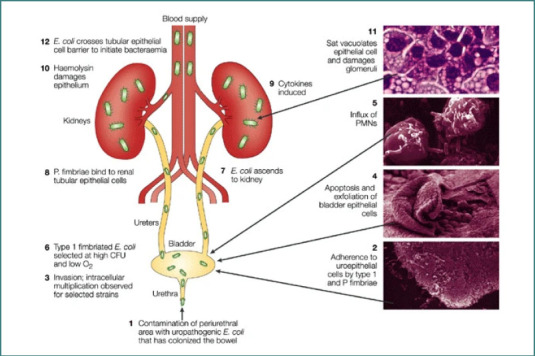
Pathogenesis of uropathogenic *Escherichia coli*. Adapted from Abdulla GM, Mohammed SA, Khedhir NH, Abed QJO, Ghareeb OA. Pathogenic bacteria caused urinary tract infections: a review. Int J Health Med Res. 2024;3(12):844-849. doi:10.58806/ijhmr.2024.v3i12n02

The high variability of the O antigen, evidenced by the existence of 186 recognized serotypes, reflects its importance in adaptation to diverse environments and hosts. Among these serotypes, uropathogenic *E. coli* (UPEC) isolates are predominantly associated with the following O serogroups: O1, O2, O4, O6, O7, O8, O14, O15, O16, O18, O21, O22, O25, O75, and O83 [30]. UPEC, classified as an ExPEC, is linked to several specific serotype combinations, including O1:H4, O1:H6, O1:H7, O1:H–, O2:H1, O2:H4, O4:H5, O6:H1, O7:H6, O7:H–, O18ac: H7, O18ac: H–, O22:H1, O25:H1, O75:H5, and O75:H7, most of which belong to phylogenetic groups B2 or D [31]. A total of six ‘O’ serogroups are responsible for 75% of the UTIs (pyelonephritis and cystitis) [11]. UPEC employs a variety of virulence factors to overcome host defenses and establish infection in the urinary tract. Among these, motility and adhesion-related proteins play critical roles in the pathogen's ability to attach to host epithelial cells, resist clearance, and invade tissues, which are essential steps in pathogenesis [32]. The virulence factors of UPEC are critical for their ability to cause infections and can be categorized into bacterial cell surface virulence factors and secreted virulence factors [33]. Figure 1 illustrates the urinary tract and the pathogenesis of infection [34]. The sequential steps of UPEC colonization, invasion, intracellular persistence, and induction of host inflammatory responses are summarized in the pathogenesis model shown in Figure 2 [35].

### Cell surface virulence factors

**a) *Fimbriae (adhesins)*:**

- Type 1 fimbriae are comprised of a main structural subunit (*FimA*), several minor subunits, and the adhesin (FimH) found at the tip of the fimbriae as well sporadically throughout the shaft [36]. It mediates adhesion to mannose-containing glycoproteins on the host epithelial cells, particularly in the bladder, facilitating urinary tract colonization and initiating infection. Fimbria/adhesins also play a role in biofilm formation and cytokine induction, which contribute to tissue damage and inflammation.
- P fimbriae (Pap pili) bind to Gal(α1 4)Gal receptors found on uroepithelial cells and kidney tissues, playing an important role in ascending UTIs and pyelonephritis, enabling bacteria to reach the kidneys [34,37,38]. Unlike type 1 fimbriae, P fimbriae–mediated adherence is mannose resistant [39]. P fimbriae specifically recognize α D galactopyranosyl (1 4) β D galactopyranoside receptors and can trigger an immune response in various models [40]. The *papG* gene encodes the PapG adhesin, which exists in three allelic forms (PapG1, PapG2, and PapG3), each with distinct substrate specificities for domains of Gal Gal disaccharide-containing glycosphingolipids [41]. P-fimbriae enhance the pathogenesis of UPEC by strongly promoting their adherence to the vascular endothelium and muscular layer, in contrast to the bladder epithelial barrier. These fimbriae also result in a high inflammatory response, leading to increased severity of UTIs. Studies have reported a positive association between P-fimbriae presence and intensity of infection [42]. Anti-P-fimbriae antibodies have been detected in the serum of the infected patients [43]. However, P-fimbriae mutant in a murine model showed no defect in colonization [44]. In contrast, in a pyelonephritis model in cynomolgus monkey, *pap* mutant resulted in reduced colonization as compared to the wild type (WT) [45]. Although P-fimbriae display a subtle role in uropathogenesis, they are strongly associated with acute pyelonephritis ascending UT [46].
- F1C and S fimbrial adhesins, often expressed by *uropathogenic Escherichia coli*, are genetically homologous [47]. However, the difference in tip adhesin between these two fimbriae confers distinct adhesive properties, and thus they are considered as separate virulence entities [48]. F1C is found in 16% of UPEC compared with 10% of fecal strains [36]. S. fimbriae are prevalent and reported in 15% and 5% of UPEC and fecal *E. coli* isolates, respectively. S. fimbriae are strongly correlated with asymptomatic bacteriuria and cystitis isolates [36]. Both have been shown to adhere to epithelial and vascular endothelial cell lines derived from the lower human UT and renal cells. S fimbriae are also associated with *E. coli* strains causing sepsis, ascending UTIs, and meningitis [49].
- Afa/Dr family fimbrial adhesins have been involved in UTIs, especially in cases like gestational pyelonephritis and recurrent cystitis [50].

b) ***Flagella***, an organelle that assists bacterial movement, play a role in how different pathogenic *E. coli* strains interact with epithelial cells, allowing UPEC to ascend along the urinary tract against urine flow, and facilitate bacterial dissemination and colonization of different niches in the urinary tract [37,51]. The bacterial strains linked to pyelonephritis might penetrate renal collecting duct (CD) cells via flagellin, which functions as an invasin in this mechanism [51].

c) ***Capsular polysaccharides*** are thick capsules with hydrophilic characteristics that form a slippery coating, making it difficult for immune cells to adhere to and engulf the bacteria, which prevents phagocytosis and protects against host complement-mediated killing [37,49,52]. Some capsular polysaccharide types, such as K1 and K5, exhibit molecular imitation with tissue components, hindering an effective humoral immune response from the infected host [37,49,52].

d) ***Lipopolysaccharide (LPS)*** is known to activate host response and to induce nitric oxide and cytokine production. A potent activator of the immune system plays a complex role in infections caused by UPEC, particularly in uncomplicated UTIs. However, its contribution to more severe complications like acute renal failure and allograft injury in ascending UTIs is less straightforward and appears to involve systemic inflammatory mechanisms rather than direct local effects within the kidney [49]. LPS are complex hydrophobic and hydrophilic molecules found in the outer membrane of bacteria and are composed of:

- Lipid A, often referred to as endotoxin, which induces strong inflammatory responses by activating TLR4 on host cells, contributing to cytokine release and tissue damage.
- Core oligosaccharide, a short chain linking Lipid A to the O antigen, containing unusual residues such as 3-deoxy-D-manno-octulosonic acid (KDO) and heptoses, which contribute to structural stability and outer membrane integrity.
- O antigen (O-specific polysaccharide), a long polysaccharide chain extending outward from the bacterial surface, consisting of repeating oligosaccharide units that are highly variable across strains. This variability aids immune evasion, protects against complement-mediated killing and phagocytosis, and contributes to bacterial adaptation [34,37,52,53].

e) ***Outer membrane vesicles (OMVs)*** are nanoscale structures produced by Gram-negative bacteria. These proteoliposomes are composed of lipids, proteins, and other biomolecules derived from the bacterial outer membrane and periplasmic space, which facilitate interaction with host cells, and serve as channels for nutrient acquisition and play a role in resistance to antimicrobial peptides [37,54]. Furthermore, OMVs are critical players in bacterial pathogenesis, survival, and intercellular communication [54]. The use of OMVs as vaccine candidates has been a promising area of research, particularly for their role in improving immunogenicity against pathogens like Neisseria meningitidis. A phase II follow-up trial highlighted the enhanced potential of combining recombinant protein antigens with OMVs to boost immune responses [54]. Bacterial nutrient metabolism mediated by OMVs indeed plays a significant role in shaping bacterial behavior, such as motility and biofilm formation, which are critical for virulence. Aromatic amino acids (AAAs), including phenylalanine, are particularly intriguing in this context due to their dual role as nutrients and signaling molecules [55].

### Secreted virulence factors

a) ***Toxins***

- Hemolysin (HlyA) is a pore-forming toxin that damages host cell membranes, leading to lysis and contributing to inflammation and tissue damage in the urinary tract. UPEC secretes α hemolysin, and its expression is strongly associated with symptomatic urinary tract infections [56]. Epidemiologically, HlyA is linked to highly virulent strains and is more frequently detected in isolates from urosepsis and pyelonephritis [57,58]. The toxin results in cell lysis, resulting in the release of nutrients and growth factors such as heme-iron, which is utilized by bacteria for successful survival inside the host. A sublytic concentration of HlyA also may enhance virulence by inactivating serine/threonine kinase Akt, which is associated with cell transduction pathways and cystic cycle progression [49,52].
- Cytotoxic necrotizing factor 1 (CNF1) modulates host cell cytoskeleton, promoting bacterial invasion and persistence within host cells [37]. Unlike some of its enteric lineages, UPEC relinquishes the need for a devoted contact-dependent secretion device to inject and manipulate proteins into the host cell [56,59]. CNF1 is a 4-layer alpha/beta/beta/alpha arrangement containing a mixed beta-sheets toxin [60]. CNF1 is expressed by UPEC and *E. coli* strains associated with neonatal meningitis. Functionally, CNF1 modifies host actin cytoskeleton by constitutively activating Rho GTPases, which are key regulators of cytoskeletal dynamics, thereby facilitating bacterial invasion of endothelial cells at the blood–brain barrier [60,61]. Further, *Sat* and *Vat* toxins are the *AT* proteins, secreted by some of the UPEC strains involved in the induction of a variety of cytopathic pathways [61].

b) ***Iron acquisition systems***

Iron is essential for numerous biological processes, including respiration and DNA synthesis. Its availability in the host is tightly regulated as part of nutritional immunity, a defense strategy that limits pathogen access to essential nutrients. Iron cannot be used in its free state; its biological functionality is dependent upon its chelation to proteins either as mono/bi-nuclear species or in a more complex assembly as heme groups or part of iron-sulfur clusters. These incorporations enable iron to adopt the appropriate redox potential, spin state, and geometry required for its diverse biological functions [62]. The host restricts pathogen access to iron through nutritional immunity. Iron is sequestered by high affinity glycoproteins, including transferrin in blood and lactoferrin in mucosal secretions (e.g., saliva, tears, milk), making it unavailable to invading microbes. Notably, lactoferrin also possesses intrinsic antimicrobial properties. Furthermore, most body iron is incorporated into heme, primarily in hemoglobin and myoglobin, where it is tightly bound and inaccessible without specialized bacterial mechanisms. Therefore, UPEC evolved sophisticated strategies to counteract iron limitation encountered in the mammalian urinary tract: (i) solubilizing ferric oxides via active secretion of acids (ii) oxidizing soluble ferrous (Fe^2^^+^) iron to the less soluble ferric (Fe^3^^+^) form; and (iii) producing specialized low molecular weight iron chelators (siderophores) to actively bind ferric ions [37,63–68]. Iron is essential for numerous biological processes, including the tricarboxylic acid (TCA) cycle, respiration, oxygen transport, nitrogen fixation, hydrogen metabolism, gene regulation, and DNA synthesis [68]. Although it is essential for almost all life forms, iron is toxic at higher concentrations. Both eukaryotic and prokaryotic cells have evolved mechanisms to maintain a balance between iron scavenging and induced iron toxicity [69]. Iron acquisition is challenging because free iron concentrations in animal blood are extremely low, whereas bacteria require approximately 10^-6^ M cytoplasmic iron for normal growth [70,71]. Iron bound within hemoglobin becomes accessible to bacteria only upon erythrocyte lysis; however, once released, it is rapidly sequestered by haptoglobin, which exerts bacteriostatic effects. The remaining iron is stored intracellularly in the form of ferritin [71]. Further, iron is linked to *heme* in hemoglobin; ferritin presents a much more efficient system for iron storage. This molecule has a mineral core formed by hydrated ferric oxide containing some phosphate [71]. Furthermore, for both commensal and pathogenic bacteria inhabiting the human host, the low availability of free iron, due to sequestration by host proteins such as heme, lactoferrin, lipocalin 2, and transferrin, necessitates the use of multiple adaptive strategies to acquire iron under iron limited conditions [63].

c) ***Autotransporters (ATs)***

Autotransporters (ATs) are multifunctional proteins that facilitate the secretion of other virulence factors and play roles in adhesion, biofilm formation, and immune modulation [37,53]. *E. coli* strains express combinations of different ATs to enable them to colonize and/or establish infections in diverse niches. ATs in *E. coli* are classified into three major groups:

- Serine protease ATs of *Enterobacteriaceae* (SPATEs);
- Trimeric AT adhesins; and
- AIDA-I type AT adhesins [72].

SPATEs, belonging to the classical type Va ATs, have passenger domains sharing the same modular organization comprising a right-handed β-helix structure with an N-terminal trypsin-like module [73]. These passenger domains are autocatalytically cleaved from the bacterial cell surface, where they then bind and digest specific intracellular and extracellular host proteins to cause tissue destruction, degrade cell protective layers, and impart serum resistance. Trimeric AT adhesins (type Vc) primarily function as adhesins [73,74]. Differences in the passenger domains of ATs determine their unique functional properties and associated virulence roles. Most AT proteins remain anchored to the bacterial membrane, although some are secreted extracellularly. In the UPEC strain CFT073, approximately 11 AT encoding genes have been identified [75]. Characterized AT proteins include Sat (a secreted toxin) and antigen Ag43a (a surface-associated adhesin), which undergoes phase variation, contributes to urovirulence, and is involved in cell aggregation and intracellular biofilm formation [76]. The trimeric AT UpaG promotes adherence to human bladder uroepithelial cells, fibronectin, and laminin and also contributes to biofilm formation and cell aggregation [24]. Similarly, surface-associated AT proteins UpaB, UpaC, and UpaH play significant roles in bladder colonization in murine models [77].

### Other virulence factors

a) ***TcpC***

The toll/interleukin-1 receptor (TIR) domain-containing protein TcpC is an important virulence factor employed by UPEC to subvert the host immune system during UTIs. TcpC targets explicitly and disrupts the toll-like receptor (TLR) signaling pathway, which is a critical component of the innate immune response. Its interaction with myeloid differentiation primary response 88 (MyD88) interrupts downstream TLR signaling, which is a key player in the innate immune system [52,65,78–80].

b) ***Outer membrane protease T (OmpT)***

Outer membrane protease T (OmpT) is another critical virulence factor produced by UPEC. One of its key functions is the activation of plasminogen to plasmin, a process that plays a crucial role in bacterial dissemination and immune modulation. Additionally, OmpT, along with other UPEC mechanisms, influences nuclear factor kappa light chain enhancer of activated B cells (NF κB) signaling, reducing the inflammatory response and enhancing bacterial survival and colonization within the urinary tract [52,81–84].

## HOST DEFENSE MECHANISM

The host employs multiple defense strategies to limit the development of UTIs caused by UPEC:

- ***Urine flow:*** the bulk flow of urine through the bladder and the process of micturition are essential mechanisms in the body's defense against UTIs by flushing away the nonattached and/or weakly attached microbes from the bladder surface. Thus, the urinary system reduces the risk of infection and maintains a healthier environment within the bladder [85,86].
- ***Urothelial shedding:*** binding of type 1 fimbriae to uroplakin 1a initiates a host response that triggers shedding of superficial bladder epithelial cells. This exfoliation serves as a defense mechanism by removing the infected cells and attached bacteria from the bladder surface. However, it also exposes underlying epithelial cells, potentially creating new opportunities for UPEC to adhere and invade, contributing to the persistence and severity of the infection [86,87].
- ***Cytokine production:*** the infection triggers the release of pro-inflammatory cytokines, such as interleukin-6 (IL-6) and interleukin-8 (IL-8), which recruit neutrophils and other immune cells to the site of infection [88,89].
- ***Antimicrobial peptides:*** the bladder epithelium secretes antimicrobial peptides, such as defensins and cathelicidins, which exert direct bactericidal effects against UPEC and inhibit bacterial growth [90].
- ***Innate immune response:*** the initial defense against UPEC involves innate immune mechanisms, including the recognition of bacterial components through pattern recognition receptors (PRRs) like TLRs [78,79].
- ***Adaptive immune response:*** in some cases, adaptive immunity is also involved, with the development of specific antibodies against UPEC antigens that can protect against recurrent infections [91].

## ANTIBACTERIAL RESISTANCE TO UPEC

In recent decades, the increased incidence and spectrum of antimicrobial-resistant UPEC, particularly multidrug-resistant (MDR) UPEC, resulted in a major clinical challenge, especially among women with recurrent UTIs and hospitalized patients, who are at higher risk of complications [92]. The spectrum of antibacterial resistance associated with UPEC is notably higher in developing countries compared to developed countries. This disparity poses significant challenges for managing UTIs globally. For instance, resistance to amoxicillin clavulanic acid in developed countries ranges from 3.1% to 40%, whereas in developing countries it is substantially higher, between 48% and 83%. Ciprofloxacin resistance rates are similarly elevated, ranging from 5.1% to 39.8% in developed countries compared with 55.5% to 85.5% in developing nations. Resistance to trimethoprim sulfamethoxazole is reported at 14.6% to 37.1% in developed regions, versus 54% to 82% in developing regions [93]. The high prevalence of MDR-UPEC in developing countries: 49.8% in Iran [94], 68% in Pakistan [95], and a staggering 98% in Mexico [96] highlights a significant public health concern, which underscores the importance of enhanced surveillance, the implementation of effective antimicrobial stewardship programs, and the need for global cooperation to manage and reduce the spread of antibiotic resistance. A large scale systematic review and meta analysis reported the highest resistance rates among UPEC to tetracyclines (69.1%), sulfonamides (59.3%), quinolones (49.4%), β lactams (36.9%), aminoglycosides (28.7%), nitrofurans (20.0%), and fosfomycin (8.4%) [97]. These high rates of resistance highlight the significant challenge posed by drug-resistant UPEC. The high resistance to tetracycline and sulfonamides, in particular, suggests the need for careful selection of antibiotics based on local resistance patterns. The relatively lower resistance to nitrofurans and fosfomycin indicates their potential continued utility as treatment options, though ongoing surveillance and stewardship are crucial to prevent the emergence and spread of resistant strains. The higher resistance rates in developing countries can be attributed to several factors, including: limited access to healthcare, inadequate antibiotic stewardship, over-the-counter antibiotic sales, and poor infection control practices [93,98–101].

The UPEC have intrinsic resistance to many hydrophobic and larger antimicrobial agents, largely due to two mechanisms:

- The outer membrane contains porins, selective channels that permit the passage of small, hydrophilic molecules while restricting larger or hydrophobic compounds. This selective permeability reduces the effectiveness of certain antibiotics, particularly those that rely on permeating the bacterial cell to exert their effect. As a result, antibiotics that are unable to pass through these porins are less effective against UPEC, contributing to its intrinsic resistance and complicating treatment strategies.
- UPEC expresses efflux pumps, which are transporter proteins that actively expel toxic substances, including antibiotics, from the cell. These pumps reduce the intracellular concentration of antibiotics, thereby diminishing their efficacy. The AcrAB-TolC efflux pump system, for example, is one of the well-characterized efflux systems in *E. coli* and plays a significant role in multidrug resistance [102].

In addition to intrinsic mechanisms, acquired antibiotic resistance in UPEC frequently arises due to antibiotic overuse and misuse in humans and involves several processes:

- ***Gene acquisition:*** Horizontal gene transfer via conjugation, transformation, or transduction enables the acquisition of resistance genes from other organisms.
- ***Mutations:*** Spontaneous mutations in bacterial DNA can lead to resistance by altering the target sites of antibiotics, modifying metabolic pathways, or enhancing efflux pump expression.
- ***Efflux pump overexpression:*** While intrinsic to some extent, efflux pumps can be overexpressed in response to antibiotic exposure, leading to increased expulsion of a broader range of antibiotics.
- ***Enzymatic degradation:*** Bacteria can produce enzymes such as beta-lactamases, which break down antibiotics like penicillin and cephalosporins, rendering them ineffective.
- ***Modification of target sites:*** bacteria can alter antibiotic target sites, such as ribosomal proteins or enzymes involved in cell wall synthesis, reducing the binding efficacy of the antibiotic
- ***Biofilm formation:*** Bacteria in biofilms are more resistant to antibiotics due to the protective matrix that limits antibiotic penetration and enhances the survival of bacterial communities [103]. Virulence-based differences in antibacterial resistance highlight the relationship between a pathogen's ability to cause disease and its mechanisms for resisting antibiotics [30].

## CONCLUSION

UTIs remain a major global public health challenge, with *Escherichia coli* responsible for approximately 70–90% of cases. This review provides a comprehensive overview of the virulence factors of UPEC and their role in UTI pathogenesis. Surface-associated factors, including adhesins, flagella, capsular polysaccharides, lipopolysaccharide, and outer membrane vesicles, along with secreted products such as toxins, iron acquisition systems, and autotransporters, as well as specialized effectors like the Toll/interleukin-1 receptor domain-containing protein TcpC and outer membrane protease T, collectively enable UPEC to adhere, invade, evade immune defenses, and persist within the urinary tract. Host defense mechanisms countering these processes include urine flow, urothelial shedding, cytokine production, antimicrobial peptides, and adaptive immune responses. It also details the general mechanisms by which bacteria develop resistance, such as gene acquisition, mutations, overexpression of efflux pumps, enzymatic degradation, biofilm formation, and modification of target sites. Although the primary focus of this review is on the mechanisms of UPEC infection, its virulence factors, and their role in disease progression, it also emphasizes the host defense mechanisms against bacterial invasion and the mechanisms of antibacterial resistance. Given the high prevalence of UPEC-associated UTIs and rising antimicrobial resistance, future strategies should focus on vaccine development, identification of novel therapeutic targets, and early diagnostic methods. Emerging technologies, including CRISPR-Cas9 systems and bioinformatics approaches, hold promise for elucidating UPEC pathogenesis and supporting the development of innovative interventions.
